# Supplementary material for: Interleukin6 prediction of mortality in critically ill COVID19 patients: A prospective observational cohort study
Source: PLoS One. 2023 Mar 1;18(3):e0279935. doi: 10.1371/journal.pone.0279935 (PMC9977034; doi:10.1371/journal.pone.0279935)
Supplement: Attachment — Submitted filename: Rebuttal letter.docx [file pone.0279935.s002.docx]

Journal Requirements:

When submitting your revision, we need you to address these additional requirements.

1. Please ensure that your manuscript meets PLOS ONE's style requirements, including those for file naming. The PLOSONE style templates can be found at

https://journals.plos.org/plosone/s/file?id=wjVg/PLOSOne_formatting_sample_main_body.pdf and

<https://journals.plos.org/plosone/s/file?id=ba62/PLOSOne_formatting_sample_title_authors_affiliations.pdf>.

OK, the new PLOSONE style template was used and the manuscript modified accordingly.

2. In the ethics statement in the Methods, you have specified that verbal consent was obtained. Please provide additional details regarding how this consent was documented and witnessed, and state whether this was approved by the IRB.

All data used in the analysis were collected in the routine surveillance activities suggested during COVID-19 pandemic, so did not require informed consent. Patients only agreed to study results dissemination. It was indeed approved by the IRB.

3. We note that you have indicated that data from this study are available upon request. PLOS only allows data to be available upon request if there are legal or ethical restrictions on sharing data publicly. For information on unacceptable data access restrictions, please see <http://journals.plos.org/plosone/s/data-availability#loc-unacceptable-data-accessrestrictions>.

OK, the data statement is modified.

4. We note you have included a table to which you do not refer in the text of your manuscript. Please ensure that you refer to Table 5 in your text; if accepted, production will need this reference to link the reader to the Table.

OK, the table 5 is now referenced in the text.

5. Please review your reference list to ensure that it is complete and correct. If you have cited papers that have been retracted, please include the rationale for doing so in the manuscript text, or remove these references and replace them with relevant current references. Any changes to the reference list should be mentioned in the rebuttal letter that accompanies your revised manuscript. If you need to cite a retracted article, indicate the article’s retracted status in the References list and also include a citation and full reference for the retraction notice.

OK

6. We noticed you have some minor occurrence of overlapping text with the following previous publication(s), which needs to be addressed:

- <https://pubmed.ncbi.nlm.nih.gov/32845568/>

Indeed, this reference is already cited in the manuscript (Ref 3)

- https://link.springer.com/article/10.1007/s12011-020-02437-9?code=1df3a266-3ae1-4a34-8d0aa94b5b77b46a& error=cookies_not_supported

Indeed, this reference is already cited in the manuscript (Ref 15)

Sentences were slightly modified.

5. Review Comments to the Author

Reviewer #1: In this manuscript, investigators examined clinical and laboratory parameters associated with COVID19 mortality among patients admitted to the ICU in Tunisia. They found that IL-6 measured within 24 hours to the ICU predicted subsequent ICU mortality. IL-6 correlated with other biomarkers including CRP, D-dimer and zinc. Invasive mechanical ventilation also predicted subsequent ICU mortality. In general, there are some minor issues but the study is technically sound. The results are not novel because similar findings have been reported in multiple other investigations.

1) Please specify the method used to determine the cutoff from the ROCs. We selected the point on the ROC curve with the minimum distance from the left-upper corner of the unit square. It corresponds to IL-6 ≥ 47.7 pg/ml with sensitivity = 0.674 and specificity = 0.857

It may be ideal to pick a value with higher sensitivity. OK, we selected another point on the ROC curve with a distance slightly less than the first from the left-upper corner of the unit square. It corresponds to IL-6 ≥ 20 pg/ml with sensitivity = 0.837 and specificity = 0.679

This second cut-off has higher sensitivity but lower specificity.

We inserted this new value in the revised manuscript and modified the Multivariate logistic regression accordingly.

2) Please specify that the associations with IL-6 and other biomarkers is statistically significant but fairly weak. This possibly accounts for the lack of association among the other biomarkers and ICU mortality. OK, we specified in the discussion that the correlation was weak.

3) It is not specified whether the patients were mechanically ventilated within 24 hours or at any point during the hospitalization. It was already specified in ‘Management and outcome’:

Invasive mechanical ventilation was required in 44 (62 %) patients with a median initiation delay of 1 [0 - 4] days.
